# Conservative versus Invasive Approaches in Temporomandibular Disc Displacement: A Systematic Review of Randomized Controlled Clinical Trials

**DOI:** 10.3390/dj12080244

**Published:** 2024-07-31

**Authors:** Manuel Sá, Carlos Faria, Daniel Humberto Pozza

**Affiliations:** 1 Experimental Biology Unit, Department of Biomedicine, Faculty of Medicine of Porto, University of Porto, 4200-319 Porto, Portugal; manuelamandiom.sa@gmail.com; 2 Department of Surgery and Physiology, Faculty of Medicine of Porto, University of Porto, 4200-319 Porto, Portugal; carlosafaria@gmail.com; 3 Institute for Research and Innovation in Health and IBMC, University of Porto, 4200-135 Porto, Portugal

**Keywords:** temporomandibular joint disorders, orofacial pain, conservative treatment, minimally invasive surgical procedures, occlusal splints, non-steroidal anti-inflammatory agents, patient-centered care, functional recovery

## Abstract

Background: Temporomandibular disorders (TMDs) frequently cause orofacial pain and dysfunction, with treatment options spanning from conservative therapies to invasive surgical procedures. The aim of this systematic review was to analyze and compare the efficacy and safety profiles of conservative, minimally invasive interventions and surgical procedures in patients diagnosed with TMDs and disc displacement. Methods: Following PRISMA recommendations, PubMed, Scopus, and Web of Science databases were searched for randomized clinical trials (RCT). Data were synthesized in a table and evaluated through the Cochrane risk of bias 2 (RoB 2) tool. Results: Thirty-eight RCTs, most with moderate RoB, were selected. Conservative approaches, including physical therapy and occlusal devices, led to an improvement in symptoms and function. Pharmacological treatments demonstrated effectiveness in reducing pain and improving function; however, they can have undesirable side effects. Minimally invasive and invasive treatments also demonstrated efficacy, although most trials did not show their superiority to conservative treatments. Conclusion: The primary approach to TMDs should be a conservative, multimodal treatment plan tailored to patient complaints and characteristics. Treatment goals should focus on symptom control and functional recovery. Surgical treatment should be reserved for cases with a precise diagnosis and a clear etiology.

## 1. Introduction

Temporomandibular disorders (TMDs) comprise a group of signs and symptoms including pain and dysfunction in the temporomandibular joint (TMJ), associated muscles, and tissues surrounding the joint. TMDs are the second most common musculoskeletal cause of pain and disability in the orofacial region [1,2,3]. According to a comprehensive meta-analysis, the global prevalence of TMDs is estimated to be around 34%. The prevalence of TMDs varies significantly across different continents. In South America, the prevalence is the highest at 47%, followed by Asia with a prevalence rate of 33%, and Europe at 29%. North America shows a prevalence rate of 25%, whereas Africa has the lowest reported prevalence at 20%. This condition predominantly affects women, with a notable male-to-female ratio of approximately 1:2 to 1:6, being higher in South America and lower in Europe. This disparity highlights the significant impact of TMDs on women’s health, making it a critical area for focused research and healthcare interventions [4].

Myogenous TMDs account for the majority of chronic orofacial pain cases [5]. Muscle pain diagnoses are classified in the Diagnostic Criteria for Temporomandibular Disorders (DC/TMD) into myalgia, tendonitis, myositis, and spasm [6]. TMDs can be caused and maintained by trauma and microtrauma. However, definitive causal factors for most cases of TMDs remain unclear [7]. A limitation of jaw movements and joint sounds such as clicking, or crepitus, are also commonly reported. The displacement of the condyle–disc system characterizes disc displacement within TMDs, where the disc is dislocated anteriorly or posteriorly to its physiologic position [7].

The Taxonomic Classification for Temporomandibular Disorders categorizes TMDs into four groups [8]: pain-related TMDs and headaches, intra-articular joint disorders, degenerative joint disorders, and subluxation. Intra-articular joint disorders can be further categorized into four distinct diagnoses: 1. disc displacement with reduction; 2. disc displacement with reduction, with intermittent locking; 3. disc displacement without reduction, with limited opening; and 4. disc displacement without reduction without limited opening. These categories are determined by observable symptoms such as jaw locking, clicking, and constraints in jaw mobility, providing a comprehensive framework for understanding and diagnosing various forms of disc displacement in TMDs [6,7,8,9]. These signs and symptoms can be aggravated during general activities like chewing [7]. Factors contributing to the development of TMDs include biomechanical stress, psychological conditions such as stress and depression, and biological factors like hormonal imbalances. Additionally, poor sleep quality and anxiety are significant contributors to TMDs. Studies have found that females are more susceptible to TMDs, and other influential factors include somatization, oral parafunction, and anatomical variations. Hypertension, higher BMI, and autoimmune disorders such as Hashimoto’s thyroiditis and SAPHO syndrome have also been linked to the increased risk of developing TMDs [4,10,11,12,13].

Studies have shown that, in many cases, TMDs resolve over time without treatment. Therefore, priority should be given to reversible, non-invasive procedures. The treatment goal should be managing symptoms and improving quality of life [14].

Conservative treatment modalities include patient education, self-management, and biobehavioral therapy. Additionally, the pharmacological approach includes short-term treatments such as NSAIDs, analgesics, benzodiazepines, muscle relaxants, antidepressants, or glucosamine and chondroitin. Physical therapy often involves the use of physical agents, including heat and cold therapy to increase circulation and reduce inflammation, respectively, electrotherapy like TENS for nerve stimulation, ultrasound for tissue healing, muscle relaxation, exercise therapy for strength and flexibility, and manual therapy for joint manipulation, among others. Each modality is chosen based on the patient’s needs, providing comprehensive care for musculoskeletal conditions and injuries. Finally, orthopedic appliances or occlusal therapy may be indicated for some patients [7,15,16,17,18].

For severe cases or those resistant to conservative treatment, surgical procedures have also been described as an option [7,19]. Surgical management may include minimally invasive procedures such as joint lavage (arthrocentesis), or more aggressive treatment including closed surgical procedures (arthroscopy) or open joint surgery (arthrotomy or arthroplasty). TMJ arthrocentesis allows for the irrigation, lavage, and/or intra-articular injection of solutions. Arthroscopy allows for the visualization of joint tissue visualization and facilitates manipulation, removal of adhesions, debridement, and biopsy [7,19].

Given that expert opinion recommends a conservative initial approach, but evidence regarding the risks and benefits of available treatment options is limited [7,19], the aim of this systematic review was to comprehensively analyze and compare the efficacy and safety profiles among various treatment modalities, ranging from conservative, minimally invasive interventions to surgical procedures, in patients diagnosed with TMDs and disc displacement.

## 2. Materials and Methods

A systematic review was undertaken following the Preferred Reporting Items for Systematic Reviews and Meta-Analyses (PRISMA) guidelines [20]. The PICO question [21] guiding this review is described in Table 1:

The inclusion criteria related to the study design comprised only randomized controlled trials (RCTs) performed in adult patients of any sex diagnosed with TMDs specifically characterized by disc displacement, with or without reduction. Only trials published in English were included. There was no restriction on the publication date; studies published up to the search date were considered. In cases where the full article was not available, efforts were made to contact the authors to obtain the full manuscript text.

Any intervention meant to treat or address symptoms of TMDs with disc displacement was assessed. Some of the interventions considered were counseling, cognitive therapy, jaw exercises, occlusal appliances, pharmacological management, arthrocentesis, arthroscopy, and arthrotomy. 

Selected outcomes include joint and masticatory muscle pain, pain pressure threshold jaw movements, maximum mouth opening, joint noises, jaw function scores, and patient quality of life.

A search was performed on 17 September 2023, using the following databases: PubMed, Scopus, and Web of Science. Search terms were developed that suited the scope of the review, adapted to each database’s query tools, and applied, filtering by publication type for randomized controlled trials. The search strategy was built up combining search words and MeSH terms. For PubMed, the search query “(“Temporomandibular Joint Disorders” [Mesh]) and “Temporomandibular Joint Disc” [Mesh]” was used; on the Scopus database, the search strategy used was “TITLE-ABS-KEY (“Temporomandibular Joint Disorders”) and “Temporomandibular Joint Disc”); and on Web of Science, we utilized the following search expression: (“Temporomandibular Joint Disorders”) and (“Temporomandibular Joint Disc”) (Title) or (“Temporomandibular Joint Disorders”) and (“Temporomandibular Joint Disc”) (Abstract) or (“Temporomandibular Joint Disorders”) and (“Temporomandibular Joint Disc”) (Author Keywords) or (“Temporomandibular Joint Disorders”) and (“Temporomandibular Joint Disc”) (Keyword Plus^®^)”. Duplicate articles were identified and removed before the screening process began.

An initial screening based on title and abstract was performed by two independent researchers during the months of September and October 2023. For the independent manuscript selection, the Rayyan tool (https://rayyan.ai/ accessed on 19 September 2023) was used in “blind mode” to ensure that reviewers were not influenced by each other’s decisions, thereby maintaining objectivity and minimizing bias in the screening process. To solve the conflict in manuscript selection, two meetings were held in November 2023 for a careful analysis and discussion to reach an agreement. Additionally, all the included manuscripts were carefully analyzed as many times as necessary to ensure appropriate inclusion. A third researcher, a specialist in TMDs, helped to resolve any doubts and reach consensus. Studies that matched our requirements were then reviewed and organized in a table in the months of November and December 2023. These studies were systematically compared and evaluated to ensure that only those with appropriate methodologies and outcomes were included in the systematic review, and that the results were valid and reliable. To ensure the process, a third researcher, a specialist in TMDs, reviewed all the information. Authors were contacted if there were missing data or no full text was available. Articles were then assessed for eligibility from the full text. The level of agreement between the authors was assessed using the kappa test [22].

## 3. Results

**Figure 1:**
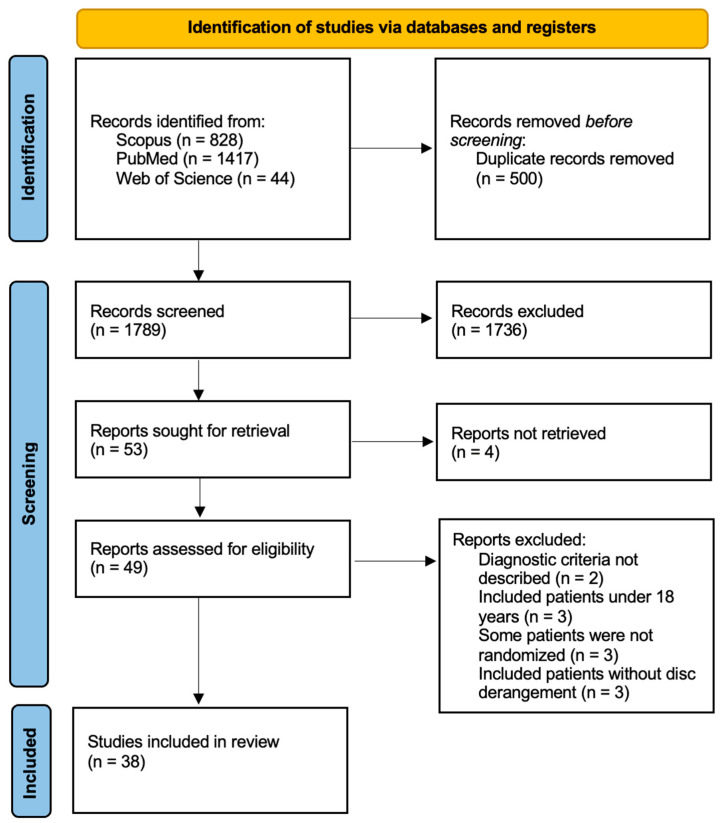
PRISMA flowchart of literature search, study screening, and inclusion.

Our initial search returned 2289 articles: 828 from Scopus, 1417 from PubMed, and 44 from Web of Science. After removing duplicate records, 1789 entries remained. Based on title and abstract, records were screened and 1736 were excluded. Of the 53 trials that were included, the researcher could not retrieve the full text of 4, which were excluded. The remaining 49 reports were assessed for eligibility. Eleven were excluded for one of the following reasons: diagnostic criteria were not described; the trial included patients under 18 years old, patient randomization was not complete, or the trial included some patients without disc derangement. A flow diagram of the selection process is depicted in Figure 1. The kappa test agreement between the authors was 0.719. Disagreement was resolved by consensus among the three authors. 

**Figure 2:**
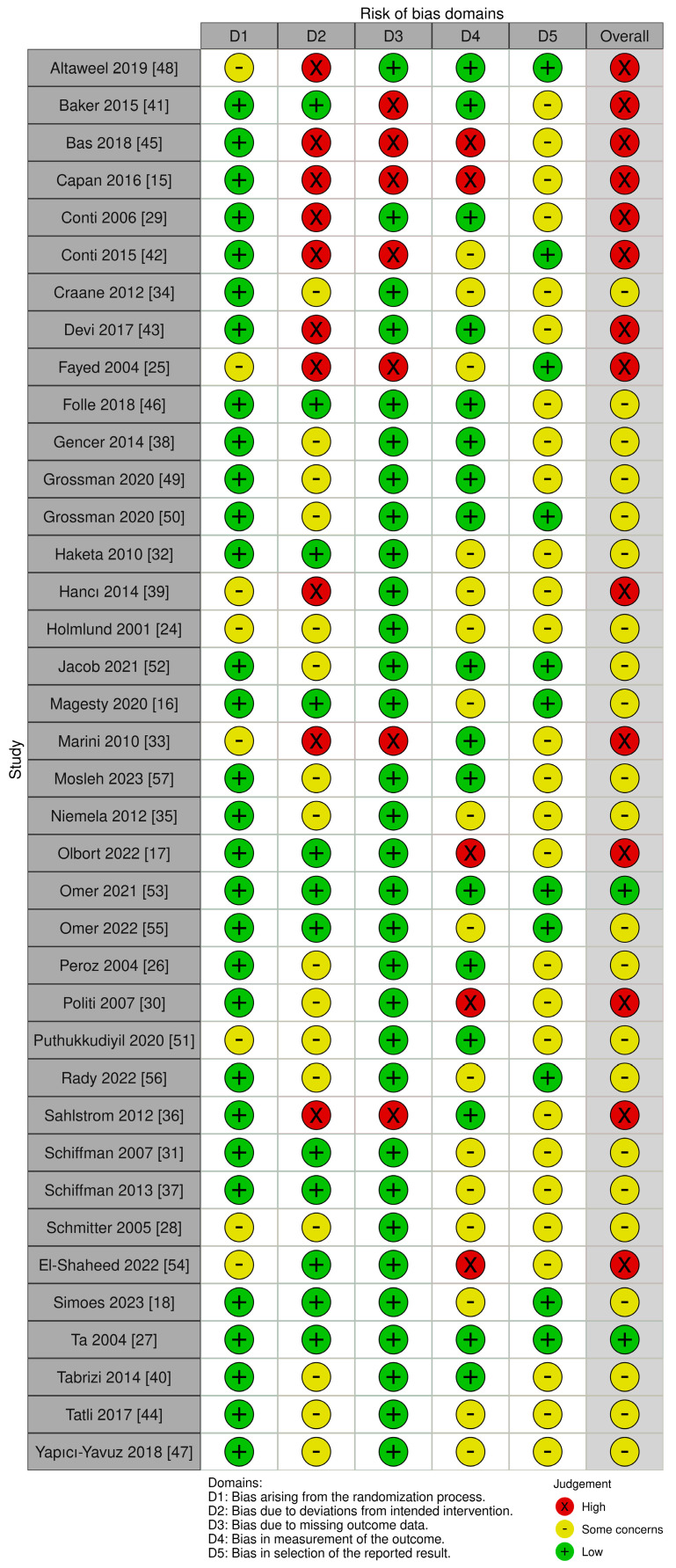
Evidence quality assessment of the studies included in this review using the TMD risk of bias tool. The risk of bias is represented in five categories and with its overall result for each study. A code of colors is used: green—low risk of bias; yellow—some concerns about bias; red—high risk of bias [15,16,17,18,24,25,26,27,28,29,30,31,32,33,34,35,36,37,38,39,40,41,42,43,44,45,46,47,48,49,50,51,52,53,54,55,56,57].

The risk of bias was assessed with the Cochrane RoB 2 tool at the outcome level visualized with the Cochrane risk of bias VISualization app 4.0 [23]; the results are available in Figure 2.

### 3.1. Description of Included Studies

The extracted data characteristics for each study are available in Table 2. The 38 studies included in this review assessed the effectiveness of physical therapy (n = 8), physical agents (n = 3), pharmacological therapy (n = 3), occlusal devices (n = 15), arthrocentesis (n = 14), arthroscopic surgery (n = 4), or open TMJ surgery (n = 5). The following main results extracted from RCTs were divided according to the treatment under study.

#### 3.1.1. Physical Therapy

Simões et al. [18] conducted a trial in 70 patients with TMD diagnoses, measuring the effect of jaw exercises alongside counseling. They concluded that the intervention group had less pain (*p* = 0.041) and click discomfort (*p* < 0.001) at the 30-day follow-up and better self-perception (*p* = 0.002). Magesty et al. [16] also examined physical therapy and confirmed the benefit of a jaw exercise program alongside counseling, with reduced pain (−1.15, *p* = 0.004), psychological discomfort (−1.49, *p* < 0.001), psychological incapacity (−1.23, *p* < 0.001), and total OHIP scores (−5.21, *p* < 0.001). However, a trial conducted by Craane et al. [34] found no additional benefit from the physical therapy program alongside medical counseling.

To assess the relative benefit of a supervised exercise program versus a home-based exercise program, Capan et al. [15] conducted a trial on 29 patients. The study revealed better outcomes for the supervised program group regarding pain and MMO (*p* < 0.05).

In a trial by Bas et al. [45], a group of 27 patients with disc displacement without reduction (DDwoR) underwent arthrocentesis and occlusal appliance treatment. They were divided into two groups. The study group was assigned self-administered physiotherapy. The conclusion drawn was that while physical exercise post-arthrocentesis had no effect on the range of mouth opening, it did reduce pain levels (difference of 1.81 in VAS, *p* < 0.05).

A study by Olbort et al. [17] showed the non-inferiority of a LPM exercise program in comparison to an occlusal stabilization appliance. Additionally, Haketa et al. [32] compared a joint exercise program to a stabilization splint-based therapy, and the exercise group had a faster recovery of jaw function (*p* < 0.001).

#### 3.1.2. Physical Agents Used through Equipment

Ekici et al. [55] assessed the effectiveness of high-intensity laser therapy (HILT) and transcutaneous electrical nerve stimulation therapy (TENS) in patients with TMDs and DDwR. The authors concluded that both interventions were beneficial, pain was reduced by 48% and 25%, respectively, and MMO was increased by 24% and 10%, respectively. The HILT group demonstrated greater improvement in symptoms and function after 12 weeks (*p* < 0.05).

Ekici et al. [53] compared HILT, ultrasound therapy (US), and occlusal splint devices to counseling and home exercise in patients with DDwR. Their results indicated that HILT, US, and occlusal splints were effective treatment options in this group of patients. Pain was reduced by 0.64 (0.32), 0.66 (0.18), and 0.44 (0.40), respectively. MMO was increased by 0.19 (0.1), 0.25 (0.23), and 0.2 (0.11), respectively.

Rady et al. [56] published a trial evaluating low-level laser therapy and botulinum toxin (BTX) intra-muscular injection in patients with DDwR in comparison with an anterior repositioning appliance (ARA). Their assessment was that both LLLT and BTX injection are effective options with a faster effect than the control. BTX can be injected via an intraoral or extraoral approach. Altaweed et al. [48] compared both approaches and their results favor the intraoral method as more convenient for the patient and faster to execute, while having similar clinical results (a mean time of 10.29 ± 2.69 for the extraoral approach and 4.86 ± 1.53 for the intraoral approach).

El-Shaheed et al. [54] conducted a study comparing LLLT and SST individually as well as in combination. They concluded that both therapies were effective, and their combination provided a faster and more effective treatment. Marini et al. [33] also evaluated the effectiveness of superpulsed LLLT in patients with DDwoR and osteoarthritis. Their results support superpulsed LLLT as a viable treatment, with significantly better results than a placebo or conservative therapy (lower VAS pain score and maximum greater mouth movement (*p* = 0.0001)).

A study by Peroz et al. [26] evaluated the use of pulsed electromagnetic fields (PEMF) to treat TMJ pain and limited mobility. They found no specific treatment effects from the therapy (*p* > 0.05).

#### 3.1.3. Intra-Articular Injections

Gencer et al. [38] investigated the effects of intra-articular injections of hyaluronic acid, tenoxicam, and dexamethasone in a trial with 100 patients. Their findings indicated that hyaluronic acid had the most significant effect on pain reduction (*p* > 0.05), while tenoxicam and dexamethasone, though less potent, still exhibited effectiveness in alleviating pain.

Platelet rich plasma (PRP) is a concentrate of plasma and growth factors capable of inducing tissue remodeling and healing [58]. Consensus on PRP preparation methods is lacking, and it is known that variations can result in differences in the biological response [59]. Hancı et al. [39] compared intra-articular PRP injection to arthrocentesis in patients with DDwR and concluded that PRP was more effective in reducing symptoms (pain score 0.07 ± 0.27 in PRP and 2.76 ± 1.48 in the control) and in improving jaw opening (MMO 39.7 ± 10.39 in PRP and 36.3 ± 5.51 in the control).

Jacob et al. [52] also studied the effect of PRP and hyaluronic acid (HA) injections alongside arthroscopy. They found that both treatments were equally effective in reducing pain and improving mouth opening.

#### 3.1.4. Occlusal Appliances

The use of an occlusal appliance alongside counseling and jaw exercises was evaluated in a study by Niemelä et al. [35]. They assigned 80 patients either to a splint group or a control group. Their results did not show that the stabilization splint treatment has additional benefits over counseling and jaw exercises at the one-month follow-up. The differences between the means were as follows: laterotrusion movement right, 0.17 (−2.21–0.22); laterotrusion movement left, 0.64 (−0.33–1.60); protrusion, 0.49 (−0.20–1.18); and active maximal opening, 1.36 (−0.43–3.15), (*p* > 0.05).

Conti et al. [42] conducted a trial on 33 participants with TMJ pain and DDwR, comparing the effectiveness of two types of orthodontic device (ARS and NTI-tss). At the two-week follow-up, 100% of the patients reported feeling more comfortable wearing anterior repositioning occlusal splints compared to their initial condition, showing an improvement when compared to the control group (66.7%, *p* < 0.05).

A study by Fayed et al. [25] investigated the effectiveness of an anterior repositioning splint and canine protected splint in nine patients with DDwR. Their analysis concluded that both splints were effective in reducing pain and promoted disc recapture in 25% and 40% of the cases, respectively.

Conti et al. [29] conducted a comparison between a canine guidance splint and a maxillary stabilization splint against a non-occluding splint. Their results lead to the conclusion that both occlusal appliances were effective in treating DDwR (*p* < 0.05).

Devi et al. [43] published a trial comparing a soft splint and a centric stabilization splint to the ARA. The conclusion drawn was that the occluding splints (ARA and centric stabilization) yielded superior results compared to the soft splint (*p* < 0.05).

Schmitter et al. [28] compared the performance of a centric occlusal splint and a distraction splint among 74 participants with DDwoR. Centric occlusal splints were more effective and therefore recommended over distraction splints (confidence interval, 1.014 to 8.741, odds ratio = 2.785).

#### 3.1.5. Pharmacological Therapy

Ta L. and Dionne R. [27] conducted a placebo-controlled trial comparing the effectiveness of celecoxib and naproxen in managing TMJ pain. By the six-week follow-up, patients in the naproxen group exhibited reduced pain levels and better mouth opening compared to both the placebo and celecoxib groups (change in VAS in naproxen group 33.05 ± 9.28; 21.08 ± 8.89 in celecoxib group; and 15.34 ± 9.51 in placebo group).

#### 3.1.6. Arthrocentesis

Tatli et al. [44] randomly assigned 120 patients to three treatment protocols: SST, arthrocentesis, or SST following arthrocentesis. Their conclusions suggested that arthrocentesis was associated with a more extensive and faster improvement (*p* = 0.000). SST had no additional benefit on the effectiveness of arthrocentesis (*p* = 1.000).

Yapıcı-Yavuz et al. [47] compared the effects of methylprednisolone acetate, sodium hyaluronate, and tenoxicam administration during arthrocentesis. Their results showed that arthrocentesis alone or with methylprednisolone acetate, sodium hyaluronate, or tenoxicam were similarly effective (*p* > 0.05).

Tabrizi et al. [40] evaluated the effect of arthrocentesis with and without corticosteroid injection. No significant differences were observed between the trial and control groups. The repeated measures test demonstrated a statistically significant improvement over time for both groups in mean (SD) pain: 8.10 (0.92) in group 1 and 7.97 (0.85) in group 2 at T0; 3.30 (1.86) in group 1 and 2.60 (1.63) in group 2 one month later; and 4.33 (1.68) in group 1 and 3.6 (1.52) in group 2 at 6 months (*p* < 0.001). The same was true for MMO: 37.17 (1.17) mm in group 1 and 36.70 (1.14) mm in group 2 at T0; 39.33 (1.58) mm in group 1 and 39 (1.43) mm in group 2 one month later; and 38.93 (1.76) mm in group 1 and 38.63 (1.71) mm in group 2 after 6 months (*p* < 0.001).

Sahlsröm et al. [36] conducted a trial to assess the effect of supplementing an extra-articular local anesthetic injection with TMJ lavage. This treatment did not improve patient outcomes at the three-month follow-up, where 74% of group A and 62% of group AL reported global improvement (*p* > 0.05). A follow-up study conducted by Baker et al. [41] re-evaluated the trial participants three years post-surgery and corroborated the previous study’s findings.

Folle et al. [46] compared double puncture and single puncture arthrocentesis and concluded that both techniques were equally effective (*p* < 0.001) in pain reduction and mouth opening, with no statistical differences between techniques (*p* > 0.05).

Grossman et al. [50] conducted a trial of two-needle arthroscopy versus double-needle cannula arthroscopy on 20 patients. Both techniques were found to be equally effective and safe without difference between groups (*p* > 0.05). However, the double needle was the fastest (*p* = 0.0001).

Grossman and Poluha [49] compared two needle positioning techniques (classic and parallel positioning) and found equivalent effectiveness in both groups. Both arthrocentesis procedures significantly reduced patient pain perception and improved mandibular movements, including the maximal interincisal distance, protrusion, and laterality (*p* < 0.001). However, there were no significant differences between the groups for these variables, except for the duration of the procedure, which was significantly faster in parallel positioning (14.81 ± 1.78 min) compared to classic (20.63 ± 2.49 min, *p* < 0.001).

#### 3.1.7. Arthroscopic Surgery

In a clinical trial by Mosleh et al. [57], two procedures were compared: arthroscopic-assisted release of the lateral pterygoid muscle and arthroscopic-assisted scarification of the retrodiscal tissues. By the end of the follow-up period, the VAS scores significantly decreased in both groups (from 6.75 to 0.45 in group muscle release and from 6.50 to 1.13 in scarification; *p* < 0.001). Additionally, maximum mouth opening increased to 32.95 ± 1.69 mm in muscle release and 30.49 ± 0.93 mm in scarification (*p* < 0.001). Both groups also showed significant improvement in lateral excursion (*p* < 0.001), and clicking sounds were eliminated in all patients. The results indicate the effectiveness of both treatments in alleviating symptoms and repositioning the TMJ disc.

#### 3.1.8. Open TMJ Surgery

Schiffman et al. [31] conducted a study assessing the effectiveness of four treatment strategies in patients with DDwoR: medical management, rehabilitation, arthroscopy with rehabilitation, and arthroplasty with rehabilitation. No differences were found between groups, leading to the recommendation of medical management and rehabilitation. A follow-up study [37] reassessed patient outcomes under different guidelines and confirmed the previous study’s conclusions. There was a 77.6% overall success rate after 60 months of self-reporting of treatment success (66/85 patients, *p* = 0.084).

A study comparing open TMJ surgery with arthroscopy was conducted by Politi et al. [30] on 20 patients with DDwoR. Similar effectiveness was found in both techniques (severity of pain intensity was significantly reduced in open surgery, and mandibular function improved with mean MFIQ score less than 7, all comparisons being statistically significant, *p* = 0.005), with arthroscopy recommend since it is the less invasive option.

Discectomy and arthroscopy were compared in a trial by Holmlund et al. [24]. A group of 22 patients were assigned to discectomy or arthroscopic lysis and lavage, and their clinical and imagological outcomes analyzed. It was demonstrated that both procedures were effective in reducing pain and dysfunction (discectomy, *p* < 0.001; arthroscopy, *p* = 0.002), and therefore the authors recommended arthroscopy as the less invasive procedure.

Puthukkudiyil et al. [51] conducted a comparison between two disc-plication procedures. According to the group allocation, 14 participants underwent treatment with either a conventional discopexy or a discopexy with bone anchors. The study revealed a more significant improvement in pain (4.57 ± 1.61 vs. 3.28 ± 0.75; *p* < 0.05) and mouth opening (14.42 ± 5.96 vs. 7.57 ± 7.25 mm; *p* < 0.05) among individuals in the bone anchor group.

## 4. Discussion

The primary findings of this systematic review demonstrated that, in most studies analyzed, conservative therapies were equally effective as surgical interventions in treating disc displacement. Furthermore, the results advocate for prioritizing conservative, less invasive treatments. Counseling was shown to be effective for most patients. Multiple studies provided support for the inclusion of jaw exercises alongside medical management and counseling. Supervised exercises also demonstrated superiority when compared to a home-based program [15]. It is important to provide proper counseling tailored to each case, as there is an abundance of information available online. Patients may misuse this information, potentially exacerbating their condition and leading them to believe that more invasive treatments are the best solution.

Conservative approaches using high-intensity laser therapy was shown to be safe and effective in reducing pain [55]. Likewise, low-level laser therapy also demonstrated its benefit in the treatment of disc displacement, with greater improvement alongside an occlusal splint device in one trial [54]. Consideration should be given to the use of laser technology due to the expenses associated with the equipment, particularly the high-intensity ones. Moreover, mastering appropriate protocols for their usage often involves a learning curve, as these protocols frequently need to be tailored to address individual patient complaints.

Occlusal appliances were more effective than non-occluding appliances [43]. When comparing distinct types of occluding appliances, most studies found no difference between groups. However, a trial found an occlusal centric splint to be superior to a distraction appliance in patients with DDwoR [28]. In general, occlusal appliances were effective in treating TMD symptoms and dysfunction. Understanding the appropriate application of splints for each individual is especially important. Factors like bruxism may reinforce the necessity for splint usage. Literature [60] indicates that soft splints may not exhibit the same efficacy as hard ones. Additionally, the frequency of use plays a significant role, with some authors suggesting intermittent use for enhanced benefits [7,61].

It is important to notice that numerous conservative treatment options exist for TMDs. These options can, and often should, be combined or alternated throughout the treatment process. Patient preference is also an important factor in treatment success, as some individuals may respond better to certain treatments, while being more reluctant to try others. Moreover, the involvement of physiotherapists specializing in TMDs can enhance commitment to exercises. For some patients, especially those experiencing chronic pain and emotional disturbances, the assistance of mental health professionals might also be necessary. In these complex contexts, a personalized medicine approach should be considered, individualizing the treatment and including other professionals in a multidisciplinary team.

There are factors beyond the intensity of the stimulus that modulate the perceived severity and impact of pain, including emotional states, patient expectation, and patient motivation. These psychological and social factors must be evaluated and considered when evaluating a TMD patient. Introduced as part of RDC/TMD and DC/TMD, the Axis II assessment tools attempt to qualify and quantify these biobehavioral factors. These aid the medical team in determining if the patient would benefit from additional psychological care [6]. If the mental healthcare needs of a patient are neglected, the treatment is likely to fail, whether it involves conservative or more invasive approaches.

A multimodal therapy can act on distinct aspects of TMD pathophysiology and may be more effective because of a synergistic effect of its components. Combining fast-acting and long-term therapies promotes patient relief through and after treatment. It can also provide better pain control by modulating it at multiple points of its pathway. Additionally, combining traditional analgesics with other therapies can reduce the amount of medication prescribed and therefore its potential risks and undesirable side effects [62,63].

The use of analgesics, antidepressants, and other drugs may serve as an important adjuvant when warranted by the case. Alleviating patient pain can promote patient comfort and enhance the effectiveness of a rehabilitation program. A patient with well controlled pain has improved jaw mobility, is less reluctant to perform prescribed exercises or physical therapy, and is more motivated to complete treatment. Analgesics with potential for tolerance or dependence should only be prescribed if essential to the treatment. Therapeutic options, including multimodal analgesia, should be explored before prescribing narcotic medications [7].

Among the included studies, only one evaluated the effect of pharmacotherapy. It demonstrated that naproxen was effective in reducing pain and improving mouth opening. However, celecoxib was not more effective than a placebo, and the authors recommend against the use of COX-2 selective inhibitors in TMDs [27,28]. The primary objective of employing analgesics, preferably in conjunction with non-pharmacological methods, is to manage acute pain effectively and to give comfort to the patient in the first approach. Analgesia is also important to prevent acute pain progression into a chronic state, which will be more challenging to manage [64,65]. However, further trials are necessary to better characterize the effect of NSAIDs and other analgesics, whether used alone or in combinations, in TMDs.

Not all non-invasive treatments will be effective in the management of TMDs. One example is that pulsed electromagnetic fields were not more effective than a placebo [26]. Despite their non-invasive nature, the limited efficacy observed in these treatments raises questions about their suitability for addressing this aspect of TMDs.

Among minimally invasive treatments, a botulinum toxin injection was shown to be superior to an anterior repositioning appliance and equivalent to LLLT. However, this was the result of a single trial [56] and should be further evaluated in future trials compared to other treatment modalities. It is also important to consider the costs, training requirements, and relatively short duration of the botulinum toxin injection procedure.

Multiple trials evaluated the effectiveness of the intra-articular injection of compounds such as hyaluronic acid, platelet rich plasma, corticosteroids, or NSAIDs. Available data support platelet-rich plasma and hyaluronic acid as the most effective in reducing pain and symptoms [38,39,47,52]. NSAIDs are a cheaper and more accessible alternative to hyaluronic acid. However, they were not as effective [38] and, due to their pharmacological nature, can potentially cause adverse side effects. The administration of corticosteroids, hyaluronic acid, or tenoxicam during arthroscopy did not significantly improve results in disc displacement when compared to simple arthroscopy procedures per se [47].

On the other hand, arthrocentesis demonstrated greater effectiveness than the stabilization splint appliance [44]. However, because of the inclusion/exclusion criteria of the present review, it was not possible to find trials comparing arthrocentesis with other non-invasive treatments. The use of TMJ lavage alongside extra-articular local anesthetic injection was not associated with less pain or better jaw movement and function [36,41]. This lack of effect persisted for up to three years after surgery.

During arthrocentesis, different needle positions can be used to access the joint space. All studied procedures were found to have similar safety and effectiveness to the conventional two-needle technique [49,50]. The procedure of arthrocentesis itself, irrespective of the specific technique employed, has shown the potential to effectively alleviate disc displacement. Its mechanical action in flushing the joint and releasing adhesions within the TMJ has demonstrated benefits in repositioning the displaced disc. However, when considering cases with a degenerative process, the incorporation of adjunctive medication should be carefully considered for managing the associated inflammatory and degenerative changes within the joint. This approach aims to address not only the acute symptoms but also the underlying degenerative process, potentially optimizing the overall treatment outcome by delaying tissue degeneration and preserving joint function [66,67].

Regarding invasive surgical procedures, most trials included found no additional benefit compared to less invasive procedures such as arthrocentesis. In the literature, evidence is split on whether arthroscopy is a more effective treatment, but most studies agree that further trials and systematic reviews are required to better evaluate these techniques [68,69]. Arthrocentesis was also associated with less tissue trauma, fewer permanent joint changes, lower complexity and cost, greater availability, and quicker post-surgical recovery [68].

Surgical approaches should be reserved for very few specific cases. Of critical importance is a precise diagnosis and a multidisciplinary approach to managing chronic orofacial pain. Misdiagnosis and repeated failed treatments are common, with surgical interventions often exacerbating pain. Therefore, surgery should be a last resort, recommended only when a specific diagnosis justifies its necessity, non-surgical therapies have been ineffective, and pain and/or dysfunction are moderate to severe [19,70]. Existing evidence supports this recommendation as TMDs have a benign course and often resolve without specific treatment [14]. Moreover, the outcomes of this systematic review highlighted the effectiveness of non-invasive to minimally invasive therapies in effectively managing patients diagnosed with TMDs and disc displacement. Thus, surgical treatment should be reserved for cases with a concrete diagnosis and specific etiology, avoiding therapeutic escalation should the treatment be ineffective. Ultimately, surgical treatment is effective when it is based on a precise diagnosis and a clear etiological factor.

Among the limitations of the present systematic review, only two studies were classified as having a low risk of bias, 22 revealed some concerns, and the remaining 14 had a high risk of bias. Due to the nature of clinical and surgical procedures, most studies did not have proper blinding and/or suffered from small sample sizes. Most of the studies that calculated a required sample size included enough participants. However, most failed to reveal a difference between the treatment and control groups. This could be due to insufficient sample size and/or limited benefit of the studied interventions. Additionally, many of the trials did not include a non-treated group and therefore we could not exclude placebo effects in their comparisons. It is also important to note that the inclusion and exclusion criteria used in the present systematic review may potentially exclude clinically significant studies within the field. Metanalysis was not possible to conduct due to the heterogeneity of the included studies.

## 5. Conclusions

TMDs often improve, or even resolve, over time, and therefore the treatment protocol should prioritize non-invasive, reversible interventions. Despite limited evidence, self-management and patient education can improve TMDs. Physical therapy, particularly exercise and manual therapy, has shown promise in improving pain and function for TMD patients. Occlusal appliances can effectively manage TMDs, especially when combined with counseling and jaw exercises, but some studies show no additional benefit over simpler treatments. Pharmacological therapy can be effective in reducing acute pain. Surgical intervention can be beneficial for some patients, namely those with moderate to severe TMDs and previous unsuccessful treatment with conventional therapy. Arthrocentesis showed similar effectiveness to other more invasive surgical procedures and therefore should be preferred.

This systematic review underscores the need for more comprehensive research to address the existing gaps and limitations, such as understanding the long-term effectiveness of non-invasive treatments, identifying the most effective self-management strategies, and evaluating the comparative benefits of various occlusal appliances and surgical interventions. This will allow for clearer guidance in clinical decision-making for the treatment of TMDs and disc displacement.

## Figures and Tables

**Table 1:** PICO question.

Population	Intervention	Comparator	Outcome
Patients diagnosed with temporomandibular disorders (TMDs) and disc displacement	Disc displacement management	Comparison between different treatment groups: - Conservative treatment, - Minimally invasive treatment, - Surgical procedures	Relieve or improve symptoms for TMDs associated with disc displacement

**Table 2:** Comparative overview of clinical studies on temporomandibular disorder treatments and outcomes.

Reference, Country	Group	Age (SD)	Gender (F/M)	N	Treatment	Main Results (»)/Follow-Up (#)	Complications
Holmlund 2001, Sweden [24]	1	37 (range 22–53)	10/0	10	Discectomy	# After 1 year » Severity of pain significantly reduced in both groups » Mandibular function, MMO, and maximum protrusion increased significantly in both groups » Reduction in joint tenderness in both groups, statistically significant only for group 1	None reported
	2	32 (range 22–46)	8/2	10	Arthroscopic lysis and lavage		
Fayed 2004, Egypt [25]	1	24	2/2	4	Anterior repositioning splint	# Both splints were effective in eliminating pain and clicking # CPS was superior in returning the disk to its normal length and shape while promoting recapture # Disc recapture was 25% in the ARS group and 40% in the CPS group	None reported
	2		1/4	5	Canine protected splint		
Peroz 2004, Germany [26]	1	43.7 (14.2)	35/7	42	Placebo device over TMJ	# Most patients reported significant improvements compared to baseline # No significant differences between groups in any follow-up # In patients with anterior DDwR »No significant improvements # In patients with anterior DDwoR » Joint noises only decreased significantly after active treatment » Assisted mouth opening increased only after placebo treatment # In patients with osteoarthrosis » Restriction of daily life activities and unassisted mouth opening improved after active treatment » Intensity of limitation improved after placebo # Strong relation between time and parameter improvement	None reported
	2		30/6	36	Pulsed electromagnetic fields over TMJ		
Ta 2004, USA [27]	1	34.5 (10.2)	17/7	24	Celecoxib 100 mg BID for 6 weeks	# All evaluations: celecoxib similar to placebo in reducing TMD pain and jaw opening # After 3 weeks, naproxen reduced pain when compared to placebo # After 4 weeks, naproxen reduced pain when compared to celecoxib # At 6 weeks, naproxen group had a significant change in mandibular opening # There is no significant difference between groups in quality of life	More adverse effects: headache in the celecoxib group and GI symptoms in the naproxen group
	2	36.6 (9.3)	16/6	22	Naproxen 500 mg BID for 6 weeks		
	3	34.7 (10.7)	13/9	22	Placebo for 6 weeks		
Schmitter 2005, Germany [28]	1	___	35/3	38	Centric occlusion splint, 18 h a day for 6 months	# 1 month » Greater improvements in the centric splint group that continued » Pain of chewing decreased in a similar way for both groups » Similar improvement in function during chewing and functions other than talking # Pain during other functions decreased faster and greater in the centric splint group throughout the trial	None reported
	2	___	3/6	36	Distraction splint (5 mm caudal and anteriorly), 18 h a day for 6 months		
Conti 2006, Brazil [29]	1	28.9	55/5	60	Stabilization splint on maxillary arch	# Significant decrease in pain score for all groups, earlier with the occluding splints and more gradual in group 3 # Significantly lower pain in group 2 when compared to group 3 # Significant differences in right lateral movement between groups 2 and 3 # All groups with improvement in left lateral, protrusive movement distance, and joint sound frequency # Reduction in muscle tenderness on palpation similar for all groups, with better results in groups 1 and 2 # Occlusal splints allowed more comfort and reduction of joints sounds	None Reported
	2	31.3			Conventional splint with canine guidance on maxillary arch		
	3	29.5			Non-occluding splint on mandibular arch		
Politi 2007, Italy [30]	1	42.9 (12.8)	7/3	10	Open-surgery high condylectomy and disk repositioning	# After 1 year » Pain intensity and joint tenderness significantly reduced in both groups » Mandibular function and maximum opening significantly improved in both groups » Clicking was not significantly reduced in any group	None reported
	2	42.7 (9.6)	7/3	10	Arthroscopic lysis, lavage, and capsular stretch		
Schiffman 2007, USA [31]	1	33.7 (1.8)	26/3	29	Medical management	» All groups improved in CMI and SSI » There was no significant difference in CMI change between groups # Arthroplasty was superior to medical management after 6 months # Arthroplasty achieved full effect by 3 months, while the other groups improved throughout the whole follow-up period # Treatment compliance was inversely associated with SSI until 18 months but not later	None reported
	2	30.0 (1.7)	25/0	25	Medical management and rehabilitation		
	3	31.8 (1.7)	22/4	26	Medical management, rehabilitation, and arthroscopic surgery		
	4	31.4 (1.9)	25/1	26	Medical management, rehabilitation, and arthroplasty		1 case of temporary nerve injury
Haketa 2010, Japan [32]	1	38.6 (13.8)	21/4	25	Stabilization splint apliance	# 8 weeks » MMO with and without pain, maximum daily pain intensity, and limitation of daily functions improved significantly in both groups » MMO improved significantly in the exercise group compared to the splint group	None reported
	2	38.8 (15.2)	19/0	19	Joint mobilization self-exercise		
Marini 2010, Italy [33]	1	41.93 (11.51)	28/11	39	GaAs diode superpulsed laser	# At baseline and day 2, pain was significantly higher in group L versus the others # From day 5 to the end of study, pain decreased dramatically in group L compared to D and C # After treatment, active and passive mouth openings and lateral motions were generally higher in the L group # MRI scans revealed that 79 of the participants had intra-articular effusion that resolved only in some patients of L group	None reported
	2	36.23 (11.30)	24/6	30	Medical treatment with NSAIDS		
	3	35.90 (6.84)	22/8	30	Laser therapy with red light		
Craane 2012, Belgium [34]	1	34.7 (14)	19/0	23	PT and counseling	# For all outcome variables, there was a significant improvement over time, independent of therapy given	None reported
	2	38.5 (15.1)	19/0	26	Counseling		
Niemela 2012, Finland [35]	1	43.2 (13.3)	32/7	39	Stabilization splint, counseling, and exercises	# 1 month » Mean pain score decreased in both groups » Splint group showed improvements in the mandibular ROM » TMJ pain on palpation increased in the splint group and decreased in the control group » No statistically significant differences between groups for any outcome	None reported
	2	44.1 (13.1)	30/11	41	Counseling and exercises		
Sahlstrom 2012, Sweden [36]	1	34.1 (12.6)	18/2	20	Extra-articular local anesthetic injection and TMJ lavage	# At baseline » Group 2 had a higher SF-JFLS score than group 1 # At both follow-ups: » No differences in pain intensity at rest and during movement and CPI » Group 2 had a significant decrease in CPI, GCPS, and pain during mandibular movements # After 1 month » Group 2 had an improvement in the vertical ROM # At the 3-month evaluation » Group 2 with further improvement in the vertical ROM and pain at rest, and a lower SF-JFLS score » The number of patients consuming analgesics decreased over time	None reported
	2	35.6 (15.6)	23/2	25	Extra-articular local anesthetic injection		
Schiffman 2013, USA [37]	1	33.7 (1.8)	26/3	29	Medical management (MM)	» Relative success rates did not differ significantly between groups » Vast difference in success rates as based on patient judgement and IAOMS criteria » Significant improvement in vertical opening, lateral and protrusive ROM, mandibular function, and TMJ and jaw muscle pain frequency and intensity » Significant worsening of osseous changes over time and therefore an increase in cases of DJD	None reported
	2	30.0 (1.7)	25/0	23	MM and rehabilitation		
	3	31.8 (1.7)	22/4	23	MM, rehabilitation, and arthroscopic surgery		
	4	31.4 (1.9)	25/1	21	MM, rehabilitation, and arthroplasty		
Gencer 2014, Turkey [38]	1	36.27 (range 18–53)	14/11	25	Intra-articular tenoxicam injection	# At 1 week and 6 weeks » Groups 1 and 3 had significantly better pain scores than the control » Group 2 had significantly better pain scores when compared to other groups	None reported
	2	38.25 (range 18–53)	14/11	25	Intra-articular hyaluronic acid injection		
	3	40.50 (range 18–49)	14/11	25	Intra-articular betamethasone injection		
	4	40.41 (18–60)	13/12	25	Intra-articular saline injection		
Hancı 2014, Turkey [39]	1	27.2 (13.4)	8/2	10	Arthrocentesis+ PRP injection + splint	# At all evaluations the study group had a significant better MMO and lower pain score and pathologic joint sounds, greatest after 6 months, versus the control group # The control group had significantly reduced pain at the 1-week, 3-months, and 6-months follow-ups # 6 months: control group had increased MMO and reduction in joint sounds	None reported
	2	25.4 (1.7)	7/3	10	Arthrocentesis + splint		
Tabrizi 2014, Iran [40]	1	28 (7.17)	22/8	30	Arthrocentesis	# At 1 month and 6 months after procedure » Significant improvement in pain severity and MMO for both groups » Group 1 had a significant reduction in clicking » No difference in pain, clicking, or MMO between the 2 groups	None reported
	2	27.07 (7.42)	25/5	30	Arthrocentesis and dexamethasone		
Baker 2015, Sweden [41]	1	38.9 (15)	31/3	12	Extra-articular local anesthetic injection and TMJ lavage	# After 3 years » Pain decreased significantly in both groups » JFLS-8, emotional and global functioning improved significantly within both groups » GCPS decreased significantly in group 2 » No differences between groups were found regarding pain relief, physical or emotional functioning or global improvement	5 reported a need for additional treatments over the 3-year time period
	2			22	Extra-articular local anesthetic injection		
Conti 2015, Brazil [42]	1	38.35	58/2	12	ARS and counseling	# 2-week evaluation » Significant reduction in pain for group 1 and 2 » Group 1 reported more comfort and improvement of initial condition # 6-week evaluation » Group 1 had significantly reduced pain compared to group 3 # 3-month evaluation » Significant reduction in pain in all groups compared to baseline » Groups 1 and 3 had decreased frequency of clicking # No significant differences were found between PPT value, mandibular ROM, or number of occlusal contacts	None reported
	2	38.4		12	TNI-tss and counseling		
	3	46		9	Counseling		
Capan 2016, Turkey [15]	1	31.0 (5.9)	15/1	16	Supervised exercise program	# 2 months » Both groups showed significant improvements in MMO, protrusion, and lateral movements » MMO and protrusion significantly greater for the study group » Both groups with reduction in pain and algometry values » Study group had a significant pain reduction » Both groups had a significant improvement of quality-of-life scores	None reported
	2	32.2 (6.0)	15/0	15	Home-based exercise program		
Devi 2017, India [43]	1	27.1 (7.19)	4/6	10	Anterior Reposition Appliance	# All groups showed significant improvement over the follow-up period # CSS showed consistent clinically effective responses and more significant improvement in follow-up visits than the SS group	None Reported
	2	30.8 (10.36)	3/7	10	Centric Stabilization Splint		
	3	32.1 (15.23)	5/5	10	Soft Splint		
Tatli 2017, Turkey [44]	1	53.2 (9.4)	35/5	40	Arthrocentesis and sodium hyaluronate	# At all follow-up visits » Significant improvements in pain, MMO, movement values, and biobehavioral scores in all groups » Pain value significantly lower in groups 1 and 2 than group 3 » MMO values significantly higher in group 1 and 2 than group 3 » Pain value and MMO were similar in groups 1 and 2 » Disability scores of groups 1 and 2 were better than group 3 # After 1 month » Psychological scores of groups 1 and 2 were lower than group 3 # At the 3- and 6-month evaluations: » Pain scores of groups 1 and 2 were significantly lower than group 3 # After 6 months: » Ipsilateral and contralateral movement values of groups 1 and 2 were significantly higher than group 3 » Groups 1 and 2 reached a higher treatment success rate than group 3	Mild transient swelling of TMJ region in 2 patients Transient hemifacial paralysis in 5 patients
	2	38.9 (11.3)	39/1	40	Stabilization splint after arthrocentesis and sodium hyaluronate		Mild transient swelling of TMJ region in 3 patients Transient hemifacial paralysis in 3 patients
	3	34.8 (8.4)	33/7	40	Stabilization splint		None reported
Bas 2018, Turkey [45]	1	33 (14.85)	25/2	14	Arthrocentesis and splint	# Pain scores markedly lower for all patients # 1-week evaluation: » No difference between groups was found in pain and MMO # 1 and 3 months: » Group 2 had significantly lower pain scores than the control	None reported
	2			13	Arthrocentesis, splint, and self-administered physiotherapy		
Folle 2018, Brazil [46]	1	30.77 (7.59)	12/1	13	Double puncture arthrocentesis	# Both techniques significantly increased maximum interincisal distance and significantly reduced pain scores, with no significant differences between groups # Relation between the duration of symptoms until treatment and the before and after pain scores	None reported
	2	37.38 (10.21)	11/2	13	Single puncture type 2 arthrocentesis		
Yapıcı-Yavuz 2018, Turkey [47]	1	___	38/6	44	Arthrocentesis and sodium hyaluronate injection	# 1 month: group 2 had significantly lower tenderness at palpation # No significant differences between groups were observed regarding MMO, decrease in pain, muscle tenderness at palpation, imagological findings, or overall treatment success	None reported
	2	___			Arthrocentesis and methylprednisolone acetate injection		
	3	___			Arthrocentesis and tenoxicam		
	4	___			Arthrocentesis		
Altaweel 2019, Egypt [48]	1	22.857 (1.864)	5/2	7	Injection of BTX-A by extraoral approach under EMG guidance	# Group 1 reported greater convenience of technique than group 2 # Significant decrease in time required with the intraoral technique # No difference between groups in vertical mouth opening, pain score or TMJ clicking and tenderness # After 8 and 16 weeks, LPM activity was significantly reduced # After 24-weeks, LPM activity was significantly increased when compared to earlier follow-ups	7 reported discomfort and increased pain in the first week, 1 nasal voice tone
	2	23.714 (2.215)	5/2	7	Injection of BTX-A by intraoral approach under EMG guidance		3 reported discomfort and increased pain in the first week, 1 nasal voice tone
Grossman 2020, Brazil [49]	1	35.90 (3.00)	18/2	20	Classic two-needle arthrocentesis	# Both procedures significantly reduced the intensity of patient pain perception and improved mandibular movements # No significant difference between groups regarding all variables, except group 2, with a shorter mean duration time	None reported
	2	32.55 (2.95)	17/3	20	Two-needle arthrocentesis with parallel positioning of second needle		
Grossman 2020, Brazil [50]	1	33.26 (5.43)	15/5	10	Two needle arthrocentesis (TNA)	# Both groups had equally significantly improved maximal interincisal distance and pain # All patients had a significant improvement of protrusive and lateral movements # The DNCA technique was significantly faster to perform that the TNA procedure	2 cases of temporary and reversible paresis of the facial nerve
	2			10	Double-needle cannula arthrocentesis (DNCA)		2 cases of temporary and reversible paresis of the facial nerve
Magesty 2020, Brazil [16]	1	22.88 (7.26)	48/22	35	Counseling and jaw exercises	# 30-day evaluation » Group 1: significant decrease in six OHIP-14 subscales and total score » Group 2: significant decrease in OHIP-14 pain and social scales and total score » Significant difference between groups in pain, psychological discomfort and disability, social disability, and total score	None reported
	2			35	Counseling		
Puthukkudiyil 2020, India [51]	1	28 (9.47)	6/1	7	Discopexy with bone anchoring	# 1 day after procedure » The pain for group 1 was higher than group 2 # After 12 months » Improvement in group 1 was significantly greater than 2 » No significant difference between groups regarding lateral excursion distance	1 case of transient temporal nerve weakness
	2	34 (12.62)	5/2	7	Conventional discopexy		None reported
Jacob 2021, India [52]	1	40.56 (9.72)	12/3	16	PRP injection and arthrocentesis	# At 3 and 6 months » Significant increase in MMO for group 1 and 2 » Pain and joint sounds decrease while MMO without pain increased in all groups	Tenderness and swelling over TMJ 1 infection
	2	46.53 (19.15)	9/6	15	HA injection and arthrocentesis		None reported
	3	51.50 (12.80)	9/7	16	Arthrocentesis		
Omer 2021, Turkey [53]	1	28.58 (14.46)	24/10	34	Splint, counseling, and exercises	# At weeks 4 and 12 » All treatments had statistically significant improvements in pain, MMO, and JFLS-20 and OHIP-14 scores compared to baseline » Groups 1, 2, and 3 had significantly lower pain and higher MMO compared to group 4 # At week 4 » OHIP-14 score was significantly improved in the groups 2 and 3 compared to the group 1 # At week 12 » No differences between the treatment groups in JFLS-20 and OHIP-14 scores	None reported
	2	28.81 (12.68)	42/8	34	US on TMJ and trigger points, counseling, and exercises		
	3	31.50 (12.67)	22/10	32	HILT on TMJ and trigger points, counseling, and exercises		
	4	31.50 (12.67)	31/3	34	Counseling and home exercises		
El-Shaheed 2022, Egypt [54]	1	26.5 (6.6)	12/2	14	Stabilization splint and Laser therapy	# All groups had significant increases in MMO and reductions in pain during the trial # Significant differences in MMO and reductions in pain between SST and LLLT vs. LLLT group and SST group at all follow-ups # Statistically significant better effect for SST and LLLT vs. SST alone # Significantly shorter time required to achieve normal state in SST and LLLT vs. LLLT or SST groups	None reported
	2	26.3 (6.9)	12/2	14	Laser therapy		
	3	38.6 (13.8)	11/3	14	Stabilization Splint Therapy		
Olbort 2022, Germany [17]	1	48.0 (17.9)	16/12	30	Muscle training	# 6 months » Both groups: reduction in orofacial pain and TMJ clicking, and improvement in muscle force » No differences between groups in pain, reduction of clicking, or maximum interincisal distance	None reported
	2	50.7 (14.8)	24/6	30	Stabilization Appliance		
Omer 2022, Turkey [55]	1	33.23 (11.66)	30/04	34	Pulsed Nd: YAG Laser Therapy and exercise	# 4 weeks » Pain, MMO, and JFLS-20 and OHIP-14 scores were significantly improved in the intervention groups » Pain and MMO were significantly improved in the HILT group compared to the TENS group # 4 and 12 weeks » JFLS-20 and OHIP-14 scores were significantly improved in the HILT group compared to the TENS group	None Reported
	2	32.25 (10.60)	30/2	32	TENS and exercise		
	3	31.17 (11.28)	31/03	34	Exercise		
Rady 2022, Egypt [56]	1	24.22 (2.9)	8/1	9	Anterior repositioning appliance	# After 3 months » Pain was reduced in all groups compared to baseline » Groups 2 and 3 had a significant increase in disc position and change in condylar position » Group 2 showed the fastest recovery time, followed by group 3	BTX injection reduced contra-lateral mandibular movements
	2	23.22 (2.1)	8/1	9	Botulinum toxin Type A injection in LPM		
	3	23.22 (2.1)	9/0	9	Laser therapy		
Mosleh 2023, Egypt [57]	1	36	___	8	Arthroscopic assisted release of the LPM	» MMO increased more in group 1 than in group 2 » Lateral excursion improved in both groups » Significant reduction in pain intensity throughout the follow-ups » Clicking sounds were absent in both groups after the intervention # At 12 months » MMO substantially improved in both groups # MRI showed adequate reduction of the disc in both groups, with no significant differences between groups	None reported
	2			8	Arthroscopic assisted scarification of the retrodiscal tissues		
Simoes 2023, Brazil [18]	1	25.88 (7.26)	48/22	35	Counseling and jaw exercises	# At baseline, patients in the test group showed right-sided pain compared to the left side in five palpation points # At the 24-h evaluation, the test group had higher pain in one palpation point # At the 7-day evaluation, no statistically significant difference was found between groups # At the 30-day evaluation » The counseling group had statistically significantly higher pain on two palpation points » Significant difference in the self-perception and click discomfort between groups and in the test group compared to the baseline.	None reported
	2			35	Counseling		

SD: Standard-deviation; F: Females; M: Males; Nd:YAG: Neodymium-doped Yttrium Aluminum Garnet; HILT: High-Intensity Laser Therapy; TENS: Transcutaneous Electrical Nerve Stimulation; MMO: Maximum Mouth Opening; JFLS-20: Jaw Functional Limitation Scale-20; OHIP-14: Oral Health Impact Profile-14; US: Ultrasound; TMJ: Temporomandibular Joint; TNI-tss: Nociceptive Trigeminal Inhibition Clenching Suppression System devices; CPS: Canine Protection Splint; SST: Stabilization Splint Therapy; LLLT: Low-Level Laser Therapy; ROM: Range of Motion; PT: Physiotherapy; DDwR: Disc Displacement with Reduction; DDwoR: Disc Displacement without Reduction; SS: Soft Splint; CSS: Centric Stabilization Splint; BTX: Botulinum Toxin; LPM: Lateral Pterygoid Muscle; PRP: Platelet-Rich Plasma; SH: Sodium Hyaluronate; GCPS: Graded Chronic Pain Severity; CPI: Chronic Pain Intensity; NSAIDS: Non-Steroidal Anti-Inflammatory Drugs; EMG: Electromyography; TNA: Two Needle Arthrocentesis; DNCA: Double Needle Cannula Arthrocentesis; CMI: Craniomandibular Index; SSI: Symptom Severity Index; DJD: Degenerative Joint Disease; »: Main Results; #: Follow-up.

## Data Availability

All data within this manuscript can be replicated by adhering to the protocols described in the Section 2.

## References

[1] Lipton J.A., Ship J.A., Larach-Robinson D. (1993). Estimated prevalence and distribution of reported orofacial pain in the United States. J. Am. Dent. Assoc..

[2] Valesan L.F., Da-Cas C.D., Réus J.C., Denardin A.C.S., Garanhani R.R., Bonotto D., Januzzi E., de Souza B.D.M. (2021). Prevalence of temporomandibular joint disorders: A systematic review and meta-analysis. Clin. Oral Investig..

[3] Ohrbach R., Greene C. (2022). Temporomandibular Disorders: Priorities for Research and Care. J. Dent. Res..

[4] Zielinski G., Pajak-Zielinska B., Ginszt M. (2024). A Meta-Analysis of the Global Prevalence of Temporomandibular Disorders. J. Clin. Med..

[5] Chan N.H.Y., Ip C.K., Li D.T.S., Leung Y.Y. (2022). Diagnosis and Treatment of Myogenous Temporomandibular Disorders: A Clinical Update. Diagnostics.

[6] Schiffman E., Ohrbach R., Truelove E., Look J., Anderson G., Goulet J.P., List T., Svensson P., Gonzalez Y., Lobbezoo F. (2014). Diagnostic Criteria for Temporomandibular Disorders (DC/TMD) for Clinical and Research Applications: Recommendations of the International RDC/TMD Consortium Network* and Orofacial Pain Special Interest Group†. J. Oral. Facial. Pain Headache.

[7] de Leeuw R., Klasser G.D. (2018). Orofacial Pain: Guidelines for Assessment, Diagnosis, and Management/American Academy of Orofacial Pain.

[8] Peck C.C., Goulet J.P., Lobbezoo F., Schiffman E.L., Alstergren P., Anderson G.C., de Leeuw R., Jensen R., Michelotti A., Ohrbach R. (2014). Expanding the taxonomy of the diagnostic criteria for temporomandibular disorders. J. Oral. Rehabil..

[9] Ohrbach R.G.Y., List T., Michelotti A., Schiffman E. Diagnostic Criteria for Temporomandibular Disorders (DC/TMD) Clinical Examination Protocol. www.rdc-tmdinternational.org.

[10] Chisnoiu A.M., Picos A.M., Popa S., Chisnoiu P.D., Lascu L., Picos A., Chisnoiu R. (2015). Factors involved in the etiology of temporomandibular disorders—A literature review. Clujul. Med..

[11] Staniszewski K., Lygre H., Bifulco E., Kvinnsland S., Willassen L., Helgeland E., Berge T., Rosen A. (2018). Temporomandibular Disorders Related to Stress and HPA-Axis Regulation. Pain Res. Manag..

[12] Warzocha J., Gadomska-Krasny J., Mrowiec J. (2024). Etiologic Factors of Temporomandibular Disorders: A Systematic Review of Literature Containing Diagnostic Criteria for Temporomandibular Disorders (DC/TMD) and Research Diagnostic Criteria for Temporomandibular Disorders (RDC/TMD) from 2018 to 2022. Healthcare.

[13] Wu J.H., Lee K.T., Kuo C.Y., Cheng C.H., Chiu J.Y., Hung J.Y., Hsu C.Y., Tsai M.J. (2020). The Association between Temporomandibular Disorder and Sleep Apnea-A Nationwide Population-Based Cohort Study. Int. J. Environ. Res. Public Health.

[14] Greene C.S. (2010). Managing the care of patients with temporomandibular disorders: A new guideline for care. J. Am. Dent. Assoc..

[15] Capan N., Esmaeilzadeh S., Karan A., Dıracoglu D., Emekli U., Yıldız A., Baskent A., Aksoy C. (2017). Effect of an early supervised rehabilitation programme compared with home-based exercise after temporomandibular joint condylar discopexy: A randomized controlled trial. Int. J. Oral Maxillofac. Surg..

[16] Magesty R.A., da Silva M.A.M., Simoes C.A.S., Falci S.G.M., Douglas-de-Oliveira D.W., Goncalves P.F., Flecha O.D. (2021). Oral health-related quality of life in patients with disc displacement with reduction after counselling treatment versus counselling associated with jaw exercises. J. Oral Rehabil..

[17] Olbort C., Pfanne F., Schwahn C., Bernhardt O. (2023). Training of the lateral pterygoid muscle in the treatment of temporomandibular joint disc displacement with reduction: A randomised clinical trial. J. Oral Rehabil..

[18] Simões C.A.S.C., da Silva M.A.M., Magesty R.A., Falci S.G.M., Douglas-de-Oliveira D.W., Gonçalves P.F., Flecha O.D. (2023). Counselling treatment versus counselling associated with jaw exercises in patients with disc displacement with reduction—A single-blinded, randomized, controlled clinical trial. BMC Oral Health.

[19] Bouloux G., Koslin M.G., Ness G., Shafer D. (2017). Temporomandibular Joint Surgery. J. Oral Maxillofac. Surg..

[20] Page M.J., McKenzie J.E., Bossuyt P.M., Boutron I., Hoffmann T.C., Mulrow C.D., Shamseer L., Tetzlaff J.M., Akl E.A., Brennan S.E. (2021). The PRISMA 2020 statement: An updated guideline for reporting systematic reviews. BMJ.

[21] Aslam S., Emmanuel P. (2010). Formulating a researchable question: A critical step for facilitating good clinical research. Indian J. Sex. Transm. Dis. AIDS.

[22] McHugh M.L. (2012). Interrater reliability: The kappa statistic. Biochem. Med..

[23] McGuinness L.A., Higgins J.P.T. (2021). Risk-of-bias VISualization (robvis): An R package and Shiny web app for visualizing risk-of-bias assessments. Res. Synth. Methods.

[24] Holmlund A.B., Axelsson S., Gynther G.W. (2001). A comparison of discectomy and arthroscopic lysis and lavage for the treatment of chronic closed lock of the temporomandibular joint: A randomized outcome study. J. Oral Maxillofac. Surg..

[25] Fayed M.M., El-Mangoury N.H., El-Bokle D.N., Belal A.I. (2004). Occlusal splint therapy and magnetic resonance imaging. World J. Orthod..

[26] Peroz I., Chun Y.H., Karageorgi G., Schwerin C., Bernhardt O., Roulet J.F., Freesmeyer W.B., Meyer G., Lange K.P. (2004). A multicenter clinical trial on the use of pulsed electromagnetic fields in the treatment of temporomandibular disorders. J. Prosthet. Dent..

[27] Ta L.E., Dionne R.A. (2004). Treatment of painful temporomandibular joints with a cyclooxygenase-2 inhibitor: A randomized placebo-controlled comparison of celecoxib to naproxen. Pain.

[28] Schmitter M., Zahran M., Duc J.M., Henschel V., Rammelsberg P. (2005). Conservative therapy in patients with anterior disc displacement without reduction using 2 common splints: A randomized clinical trial. J. Oral Maxillofac. Surg..

[29] Conti P.C., dos Santos C.N., Kogawa E.M., de Castro Ferreira Conti A.C., de Araujo Cdos R. (2006). The treatment of painful temporomandibular joint clicking with oral splints: A randomized clinical trial. J. Am. Dent. Assoc..

[30] Politi M., Sembronio S., Robiony M., Costa F., Toro C., Undt G. (2007). High condylectomy and disc repositioning compared to arthroscopic lysis, lavage, and capsular stretch for the treatment of chronic closed lock of the temporomandibular joint. Oral Surg. Oral Med. Oral Pathol. Oral Radiol. Endod..

[31] Schiffman E.L., Look J.O., Hodges J.S., Swift J.Q., Decker K.L., Hathaway K.M., Templeton R.B., Fricton J.R. (2007). Randomized effectiveness study of four therapeutic strategies for TMJ closed lock. J. Dent. Res..

[32] Haketa T., Kino K., Sugisaki M., Takaoka M., Ohta T. (2010). Randomized clinical trial of treatment for TMJ disc displacement. J. Dent. Res..

[33] Marini I., Gatto M.R., Bonetti G.A. (2010). Effects of Superpulsed Low-level Laser Therapy on Temporomandibular Joint Pain. Clin. J. Pain.

[34] Craane B., Dijkstra P.U., Stappaerts K., De Laat A. (2012). Randomized controlled trial on physical therapy for TMJ closed lock. J. Dent. Res..

[35] Niemelä K., Korpela M., Raustia A., Ylöstalo P., Sipilä K. (2012). Efficacy of stabilisation splint treatment on temporomandibular disorders. J. Oral Rehabil..

[36] Sahlström L.E., Ekberg E.C., List T., Petersson A., Eriksson L. (2013). Lavage treatment of painful jaw movements at disc displacement without reduction. A randomized controlled trial in a short-term perspective. Int. J. Oral Maxillofac. Surg..

[37] Schiffman E.L., Velly A.M., Look J.O., Hodges J.S., Swift J.Q., Decker K.L., Anderson Q.N., Templeton R.B., Lenton P.A., Kang W. (2014). Effects of four treatment strategies for temporomandibular joint closed lock. Int. J. Oral Maxillofac. Surg..

[38] Gencer Z.K., Özkiriş M., Okur A., Korkmaz M., Saydam L. (2014). A comparative study on the impact of intra-articular injections of hyaluronic acid, tenoxicam and betametazon on the relief of temporomandibular joint disorder complaints. J. Cranio-Maxillofac. Surg..

[39] Hancı M., Karamese M., Tosun Z., Aktan T.M., Duman S., Savaci N. (2015). Intra-articular platelet-rich plasma injection for the treatment of temporomandibular disorders and a comparison with arthrocentesis. J. Craniomaxillofac. Surg..

[40] Tabrizi R., Karagah T., Arabion H., Soleimanpour M.R., Soleimanpour M. (2014). Outcomes of arthrocentesis for the treatment of internal derangement pain: With or without corticosteroids?. J. Craniofacial Surg..

[41] Baker Z., Eriksson L., Englesson Sahlström L., Ekberg E. (2015). Questionable effect of lavage for treatment of painful jaw movements at disc displacement without reduction: A 3-year randomised controlled follow-up. J. Oral Rehabil..

[42] Conti P.C.R., Correa A.S.D., Lauris J.R.P., Stuginski-Barbosa J. (2015). Management of painful temporomandibular joint clicking with different intraoral devices and counseling: A controlled study. J. Appl. Oral Sci..

[43] Devi J., Verma M., Gupta R. (2017). Assessment of treatment response to splint therapy and evaluation of TMJ function using joint vibration analysis in patients exhibiting TMJ disc displacement with reduction: A clinical study. Indian J. Dent. Res..

[44] Tatli U., Benlidayi M.E., Ekren O., Salimov F. (2017). Comparison of the effectiveness of three different treatment methods for temporomandibular joint disc displacement without reduction. Int. J. Oral Maxillofac. Surg..

[45] Bas B., Kazan D., Kutuk N., Gurbanov V. (2018). The Effect of Exercise on Range of Movement and Pain After Temporomandibular Joint Arthrocentesis. J. Oral Maxillofac. Surg..

[46] Folle F.S., Poluha R.L., Setogutti E.T., Grossmann E. (2018). Double puncture versus single puncture arthrocentesis for the management of unilateral temporomandibular joint disc displacement without reduction: A randomized controlled trial. J. Cranio-Maxillofac. Surg..

[47] Yapıcı-Yavuz G., Şimşek-Kaya G., Oğul H. (2018). A comparison of the effects of methylprednisolone acetate, sodium hyaluronate and tenoxicam in the treatment of non-reducing disc displacement of the temporomandibular joint. Med. Oral Patol. Oral Y Cir. Bucal.

[48] Altaweel A.A., Elsayed S.A., Baiomy A., Abdelsadek S.E., Hyder A.A. (2019). Extraoral Versus Intraoral Botulinum Toxin Type A Injection for Management of Temporomandibular Joint Disc Displacement with Reduction. J. Craniofac. Surg..

[49] Grossmann E., Poluha R.L. (2021). Comparison between TMJ arthrocentesis techniques with different needle positions: A randomized single-blind controlled clinical trial. J. Cranio-Maxillofac. Surg..

[50] Grossmann E., Ferreira L.A., Poluha R.L., Setogutti E., Iwaki L.C.V., Iwaki Filho L. (2022). Comparison of two needles arthrocentesis versus double needle cannula arthrocentesis in the treatment of temporomandibular disc displacement. Cranio J. Craniomandib. Pract..

[51] Puthukkudiyil J.S., Bhutia O., Roychoudhury A., Bhatt K., Yadav R., Bhalla A.S. (2020). Does Repositioning of Temporomandibular Joint Disc with Bone Anchors Provide Better Clinical Outcomes Than Conventional Disc Plication Procedures for Anterior Disc Displacements without Reduction in Patients Refractory to Nonsurgical Treatments?. J. Oral Maxillofac. Surg..

[52] Jacob S.M., Bandyopadhyay T.K., Chattopadhyay P.K., Parihar V.S. (2022). Efficacy of Platelet-Rich Plasma Versus Hyaluronic Acid Following Arthrocentesis for Temporomandibular Joint Disc Disorders: A Randomized Controlled Trial. J. Maxillofac. Oral Surg..

[53] Ekici Ö., Dündar Ü., Gökay G.D., Büyükbosna M. (2022). Evaluation of the efficiency of different treatment modalities in individuals with painful temporomandibular joint disc displacement with reduction: A randomised controlled clinical trial. Br. J. Oral Maxillofac. Surg..

[54] El-Shaheed N.H., Mostafa A.Z.H., Aboelez M.A. (2023). Efficacy of stabilisation splint and low-level laser therapy for patients with chronic closed lock from non-reducible displaced temporo-mandibular joint discs: A parallel randomised clinical trial. J. Oral Rehabil..

[55] Ekici Ö., Dündar Ü., Büyükbosna M. (2022). Comparison of the Efficiency of High-Intensity Laser Therapy and Transcutaneous Electrical Nerve Stimulation Therapy in Patients with Symptomatic Temporomandibular Joint Disc Displacement with Reduction. J. Oral Maxillofac. Surg..

[56] Rady N.A., Bahgat M.M., Abdel-Hamid A.M. (2022). Promising minimally invasive treatment modalities for symptomatic temporomandibular joint disc displacement with reduction: A randomized controlled clinical trial. BMC Oral Health.

[57] Mosleh A.A.E.L., Nowair I.M., Saad K.A.E.H., Sadakah A.E.F.A.E.M. (2023). Arthroscopic assisted release of lateral pterygoid versus scarification of retrodiscal tissue in management of internal derangement of temporomandibular joint-A randomized clinical trial. J. Cranio-Maxillofac. Surg..

[58] Marx R.E. (2004). Platelet-rich plasma: Evidence to support its use. J. Oral Maxillofac. Surg..

[59] Machado E.S., Soares F.P., Vianna de Abreu E., de Souza T., Meves R., Grohs H., Ambach M.A., Navani A., de Castro R.B., Pozza D.H. (2023). Systematic Review of Platelet-Rich Plasma for Low Back Pain. Biomedicines.

[60] Okeson J.P. (1987). The effects of hard and soft occlusal splints on nocturnal bruxism. J. Am. Dent. Assoc..

[61] Albagieh H., Alomran I., Binakresh A., Alhatarisha N., Almeteb M., Khalaf Y., Alqublan A., Alqahatany M. (2023). Occlusal splints-types and effectiveness in temporomandibular disorder management. Saudi Dent. J..

[62] Turk D.C., Rudy T.E., Kubinski J.A., Zaki H.S., Greco C.M. (1996). Dysfunctional patients with temporomandibular disorders: Evaluating the efficacy of a tailored treatment protocol. J. Consult. Clin. Psychol..

[63] Nguyen P., Mohamed S.E., Gardiner D., Salinas T. (2001). A randomized double-blind clinical trial of the effect of chondroitin sulfate and glucosamine hydrochloride on temporomandibular joint disorders: A pilot study. Cranio.

[64] Greene C.S., Manfredini D. (2021). Transitioning to chronic temporomandibular disorder pain: A combination of patient vulnerabilities and iatrogenesis. J. Oral Rehabil..

[65] Voscopoulos C., Lema M. (2010). When does acute pain become chronic?. Br. J. Anaesth..

[66] Clegg T.E., Caborn D., Mauffrey C. (2013). Viscosupplementation with hyaluronic acid in the treatment for cartilage lesions: A review of current evidence and future directions. Eur. J. Orthop. Surg. Traumatol..

[67] Kalladka M., Quek S., Heir G., Eliav E., Mupparapu M., Viswanath A. (2014). Temporomandibular joint osteoarthritis: Diagnosis and long-term conservative management: A topic review. J. Indian Prosthodont. Soc..

[68] Laskin D.M. (2018). Arthroscopy Versus Arthrocentesis for Treating Internal Derangements of the Temporomandibular Joint. Oral Maxillofac. Surg. Clin. North Am..

[69] Al-Moraissi E.A. (2015). Arthroscopy versus arthrocentesis in the management of internal derangement of the temporomandibular joint: A systematic review and meta-analysis. Int. J. Oral Maxillofac. Surg..

[70] Israel H.A., Ward J.D., Horrell B., Scrivani S.J. (2003). Oral and maxillofacial surgery in patients with chronic orofacial pain. J. Oral. Maxillofac. Surg..

